# *Pteris latipinna* sp. nov. (Pteridaceae), a new species segregated from *Pteris fauriei*

**DOI:** 10.3897/phytokeys.85.14884

**Published:** 2017-08-31

**Authors:** Yi-Shan Chao, Atsushi Ebihara, Wen-Liang Chiou, Yao-Moan Huang

**Affiliations:** 1 Department of Biomedical Science & Environmental Biology, Kaohsiung Medical University, 100, Shih-Chuan 1st Rd., Kaohsiung, 80708, Taiwan; 2 Department of Botany, National Museum of Nature and Science, 4-1-1, Amakubo, Tsukuba-shi, Ibaraki 305-0005, Japan; 3 Taiwan Forestry Research Institute, 53 Nan-Hai Rd., Taipei, 10066, Taiwan; 4 Dr. Cecilia Koo Botanic Conservation Center, 31, Tongsing Rd., Gaoshu Township, Pingtung County, 90646, Taiwan

**Keywords:** *Pteris*, *Pteris fauriei*, *Pteris latipinna*, Taiwan, taxonomy

## Abstract

*Pteris fauriei* is widely distributed in Eastern Asia and has high morphological variation. Some morphologically similar plants related to this species are difficult to distinguish. We showed that the new *Pteris* species from Taiwan, previously identified as *P. fauriei*, can be morphologically distinguished by its wide pinnae, larger terminal pinnae than the lateral pinnae in sterile fronds, and triangular basal segments of the lateral pinnae. It was confirmed that this species is phylogenetically separated from the other East Asian *Pteris* species, except for a morphologically distinct species *P. arisanensis*, by means of chloroplast genes, *rbcL* and *matK*. The new species is named as *Pteris latipinna* **sp. nov.**, referring to its wide pinnae. Here, we provide a key to facilitate the identification of the morphologically similar *Pteris* species in Asia. The morphological descriptions, images, ecology, and distribution are also presented.

## Introduction

**Figure 1.**
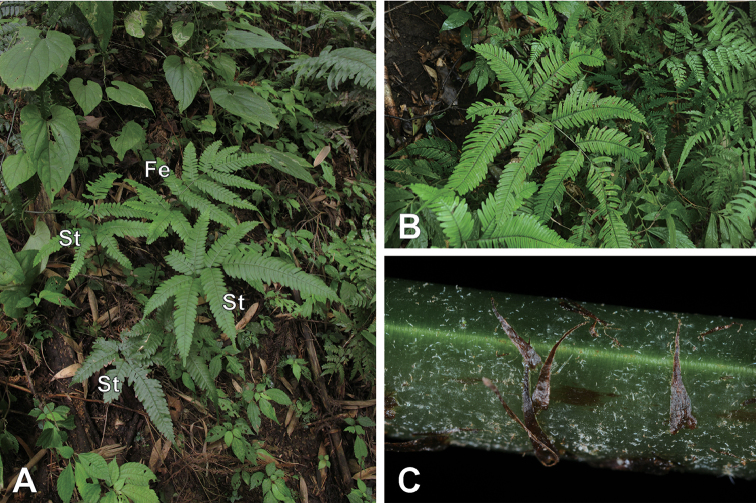
Photographs of *Pteris latipinna* Y.S.Chao & W.L.Chiou, sp. nov. in Hsinchu, Taiwan. **A** Habitat. Terminal pinna of sterile frond is larger than the lateral pinna. St, sterile fronds; Fe, fertile fronds **B** A frond **C** Concolorous scales a stipe.

*Pteris fauriei* Hieron. is widely distributed in Eastern Asia. Two varieties of *P. fauriei* have been confirmed, and both varieties have different cryptic characteristics and prefer different niches. Pteris fauriei Hieron. var. fauriei, apomictic and triploid (2n = 87), usually has herbaceous laminae and prefers cooler sites; P. fauriei var. minor Hieron., sexual and diploid (2n = 58), usually has coriaceous laminae and is found in warmer sites (Huang et al. 2006; Huang et al. 2007). In Taiwan, some undescribed *Pteris* plants (Fig. 1), usually regarded as P. fauriei var. fauriei, with herbaceous laminae were found in understory of evergreen forests. However, those plants have wider laminae and pinnae than other bipinnatifid *Pteris* species recorded in Taiwan. Outside Taiwan, *P. natiensis* Tagawa, a Japanese endemic fern (Iwatsuki 1995), apomictic and diploid (Nakato and Ebihara 2016), is the most morphologically similar species in East Asia.

*Pteris fauriei* and morphologically similar *Pteris* species are phylogenetically close. Chao et al. (2014) revealed that the clade (A1, including those species with bipinnatified laminae mostly) arose more recently than most of other clades in *Pteris*. Because of similar morphology, the characteristics to delimitate species need to be examined and compared in detail, such as venation, scale color, shapes of pinnae and segments, and pinnae stalked or sessile (Chao et al. 2013).

In this study, we clarified the morphological and phylogenetic characteristics of the undescribed *Pteris* plants, in comparison with *P. fauriei*, *P. natiensis*, and related bipinnatifid *Pteris* species from East Asia, including *P. wulaiensis* C.M. Kuo endemic to Taiwan; *P. arisanensis* Tagawa, *P. biaurita* L., *P. kawabatae* Sa. Kurata, *P. kiuschiuensis* Hieron., and *P. oshimensis* Hieron. distributed in China and Japan; and *P. boninensis* H. Ohba, *P. laurisilvicola* Sa. Kurata, *P. satsumana* Sa. Kurata, and *P. yakuinsularis* Sa. Kurata endemic to Japan (Iwatsuki 1995; Liao et al. 2013). On the basis of morphological and molecular data, the taxonomic treatments were applied.

## Materials and methods

### Morphology

We examined type materials of morphologically similar taxa, including P. fauriei var. fauriei (in herbaria B, BM, KYO, MO, P), P. fauriei var. minor (in herbaria B, BM, KYO, P), and *P. natiensis* (in herbaria KYO, P). Several morphologically similar species in neighboring areas were also compared, including *P. arisanensis*, *P. biaurita*, *P. boninensis*, *P. kawabatae*, *P. kiuschiuensis*, *P. laurisilvicola*, *P. oshimensis*, *P. satsumana*, *P. wulaiensis*, and *P. yakuinsularis*.

### Phylogenetic analyses

To clarify the phylogenetic relationships of the undescribed plants, 34 other *Pteris* taxa with bipinnatifid laminae were sampled. Three *Pteris* species, *P. grevilleana*, *P. longipinna*, and *P. venusta*, were used as outgroups. These bipinnatifid and outgroup species belong to clades A1 and A2, respectively, according to the phylogenetic tree of *Pteris* (Chao et al. 2014). Vouchers and GenBank accession numbers are listed in Appendix 1. Total genomic DNA was extracted from young fronds, following a modified cetyltrimethylammonium bromide (CTAB) method (Doyle and Doyle 1990). Two chloroplast genes, *rbcL* and *matK*, were amplified using the PCR primers for *rbcL* and *matK* as per Chao et al. (2014). Alignment was performed with ClustalW (Thompson et al. 1994) and manually edited using BioEdit 7.1.3 (Hall 1999). Gaps were treated as missing data.

Maximum likelihood (ML) analyses were performed using GARLI v.2.0.1019 (Zwickl 2006). Ten independent runs were conducted using automatic termination following 10,000 generations without a significant (lnL increase of 0.01) change in topology. To calculate ML bootstrap support for each node, 1,000 bootstrap replicates were performed with automatic termination at 10,000 generations, under one run.

## Results

### Morphology

The distinct morphologies that distinguished the undescribed species from other bipinnatifid *Pteris* species are its wide pinnae, up to 7 cm wide, and fewer pairs of lateral pinnae, only 2–5 pairs (Fig. 1). Furthermore, its terminal pinnae of sterile fronds are larger than the lateral pinnae (Table 1). In Taiwan, these characteristics can separate the undescribed species from P. fauriei var. fauriei and P. fauriei var. minor (these two taxa were illustrated by one of their type materials, Figs S1 and S2, respectively).

An endemic species in Japan, *Pteris natiensis* (illustrated by holotype, KYO, Fig. S3), also has sterile fronds with slightly larger terminal pinnae than the lateral pinnae. Its pinnae are slightly narrower than those of the undescribed species (3–5 cm vs. 3–7 cm), and the basal pinna-segments are adnate to the rachis whereas they are not adnate to the rachis in the undescribed species (Table 1). Another specific trait of the undescribed species is the triangular (vs. falcate) basal segments of the lateral pinnae, which could be used to identify the new species from other similar species, including *P. fauriei* and *P. natiensis* (Table 1). The triangular and falcate basal segments are resulted by the longer costa adnate with the segments of the undescribed species and shorter costa adnate with the segments of the other species, respectively.

**Table 1.** Morphological comparisons among *Pteris latipinna* Y.S.Chao & W.L.Chiou, sp. nov., P. fauriei var. fauriei, P. fauriei var. minor, and *P. natiensis*.

Species/Characteristics	*P. latipinna*	P. fauriei var. fauriei	P. fauriei var. minor	*P. natiensis*
Lamina size	15–45 cm long, 15–40 cm wide; length/width ratio about 1	15–40 cm long, 10–35 cm wide; length/width ratio 1.2–1.5	10–30 cm long, 10–25 cm wide; length/width ratio about 1	15–40 cm long, 10–35 cm wide; length/width ratio about 1.1–1.2
Number of lateral pinnae of sterile fronds	2–3(4) pairs	2–7 pairs	2–5 pairs	2–5 pairs
Lateral pinnae of sterile fronds	Slightly incurved	Straight	Straight	Incurved
Petiolule	Sessile or short-petiolate. Most basal pinna-segments free to the rachis, sometimes adnate	Sessile or short-petiolate. Basal pinna-segments free to the rachis	Sessile or short-petiolate. Basal pinna-segments free to the rachis	Sessile. Basal pinna-segments adnate to the rachis; except basal pinnae
Basal segment of lateral pinnae	Triangular	Falcate	Falcate	Falcate
Terminal pinna size of sterile fronds	Distinctly wider than lateral pinnae except basal ones	Smaller than lateral pinnae	Smaller than lateral pinnae	Almost the same size as lateral pinnae
Pinna shape	Ovate-lanceolate, distinctly narrowed at base	Lanceolate, not narrowed at base	Lanceolate, not narrowed at base	Ovate to lanceolate, more and less narrowed at base
Width of lateral pinna	3–7 cm	2–3.5 cm	1–3 cm	3–5 cm

### Phylogeny and chloroplast DNA differences

Genetic data and the accession numbers of the sequences are listed in Appendix 1. The chloroplast DNA (cpDNA) alignment matrix of *rbcL* (1,278 bp) and *matK* (900 bp) contained a total of 2,178 characters with 121 parsimony-informative sites. The log-likelihood score for the most likely ML tree was -5304.42470.

**Figure 2.**
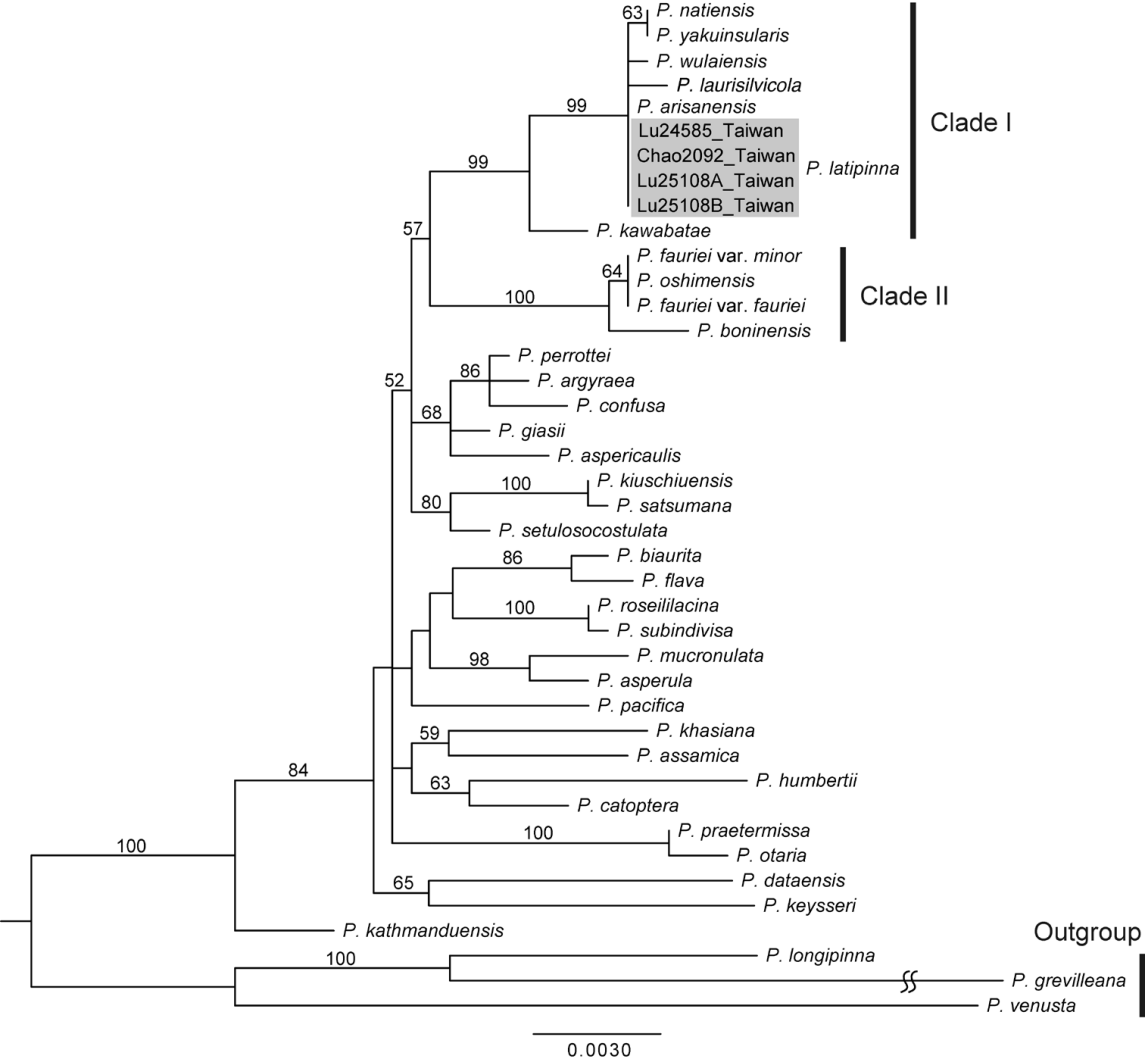
Chloroplast DNA phylogeny of the *Pteris latipinna* Y.S.Chao & W.L.Chiou, sp. nov. and related taxa. ML bootstrap support values are indicated on each branch.

The phylogenetic tree (Fig. 2) infers that the *Pteris* species with bipinnatifid laminae formed one monophyletic group (the clade of ingroup taxa), as revealed in the previous *Pteris* phylogeny (Chao et al. 2014). The undescribed taxon and *P. fauriei* were divided into two different clades, Clade I and II. In Clade I, the undescribed taxon shared identical cpDNA sequences with *P. arisanensis*, although they can be separated by their morphologies, such as venation and lamina shape (Fig. S4). The undescribed taxon cpDNA differed from *P. natiensis*, *P. wulaiensis* (Fig. S5), and *P. yakuinsularis* cpDNA by one nucleotide substitution, and from *P. laurisilvicola* cpDNA by two nucleotide substitutions. In Clade II, P. fauriei var. fauriei, P. fauriei var. minor, and *P. oshimensis* shared identical cpDNA sequences.

**Figure 3.**
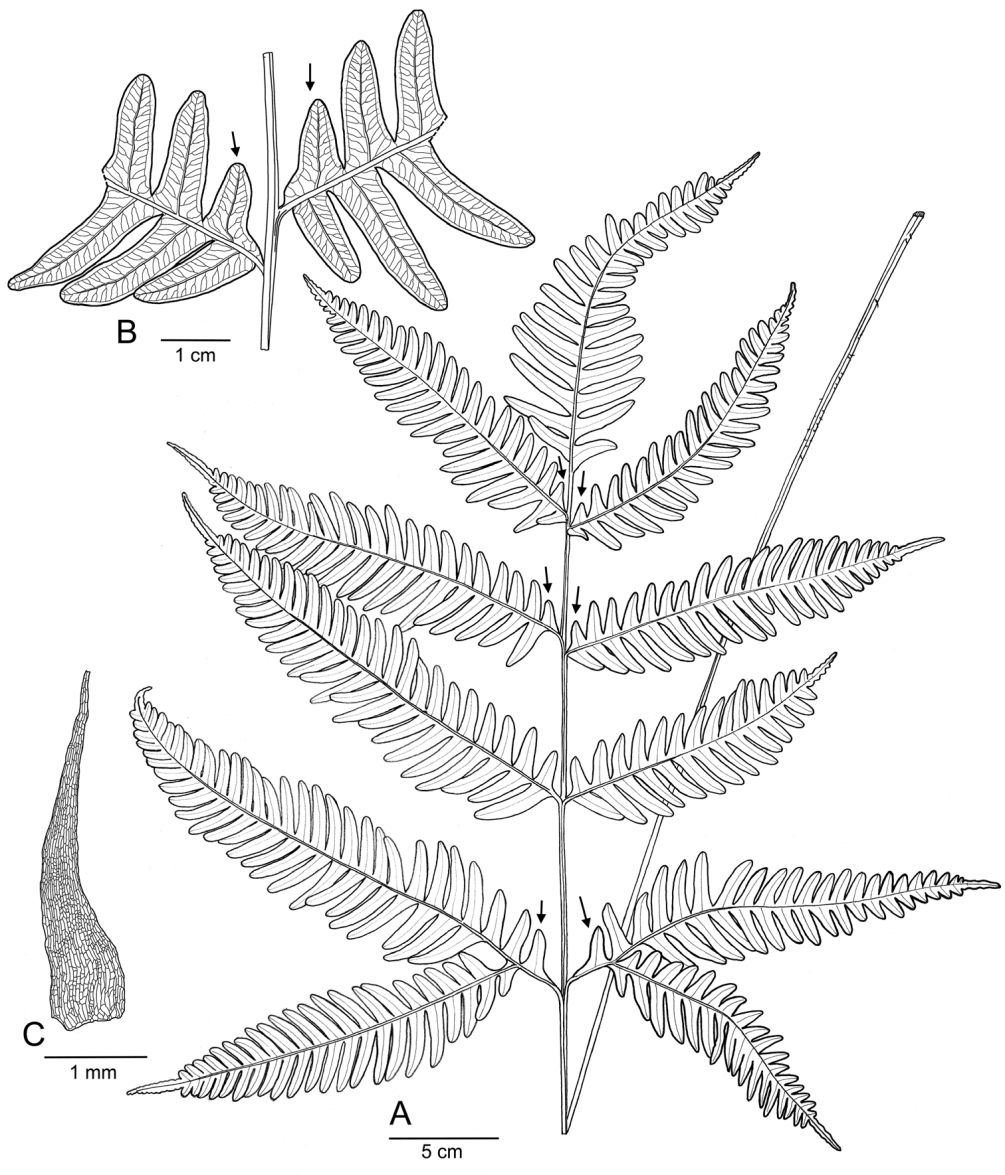
Illustration of *Pteris latipinna* Y.S.Chao & W.L.Chiou, sp. nov., based on holotype. **A** A fertile frond **B** Venation **C** Linear, concolorous scale. Basal segments of lateral pinnae are triangular (indicated by arrows).

Both morphological and DNA characteristics support that this taxon is a new species, rather than a variety of *P. fauriei*. Here, we describe the new species and delimitate P. fauriei var. fauriei and P. fauriei var. minor. The morphology of the new species is presented in Fig. 3 and described below.

### Taxonomic treatment

#### Pteris latipinna

Taxon classificationPlantaePolypodialesPteridaceae

Y.S.Chao & W.L.Chiou sp. nov.

urn:lsid:ipni.org:names:77165362-1

Figs 1 , 3

##### Type.

TAIWAN. Hsinchu County: Zhudong Town, Wuchihshan, 3 March 2013, *Y.-S. Chao 2092* (holotype TAIF!, isotype TAIF!, TNS!).

##### Description.

Rhizomes short, ascending, apex scaly; scales linear lanceolate, 1–4 mm long, 0.2–0.5 mm wide, concolorous, dark brown, entire, apex long-acuminate. Fronds clustered, 30–100 cm long, nearly monomorphic. Sterile fronds 30–70 cm long; stipes green, 2–4 mm thick, 10–30 cm long, base with persistent and scattered scales; grooved on the adaxial side; laminae widely ovate, 15–45cm long, 15–40 cm wide, bipinnatifid; 2–3(4) pairs lateral pinnae, pinna angle against rachis 60–70°, straight, basal pinnae with one pair of exaggerated basiscopic pinnules, terminal pinnae distinctly longer and wider than the lateral except basal ones; pinnae ovate-lanceolate, distinctly narrowed at base, pectinate, 8–21 cm long, 3–7 cm wide, sessile or short-petiolate, apex caudate, 1–4 cm long. Basal segments of the lateral pinnae triangular, the other segments of pinnae falcate, 4–9 mm wide, apex obtuse, margins entire; veins forked, free. Fertile fronds 50–105 cm long; stipes 25–55 cm long; laminae ovate to widely ovate, 20–50 cm long, 20–35cm wide, bipinnatifid; 3–5 pairs lateral pinnae, slightly incurved or straight; terminal pinna usually wider than the lateral; pinnae 8–20 cm long, 2–6 cm wide, 1–4 cm long; segments of pinnae 4–6 mm wide, apex acute or obtuse. Sori along pinna margins, protected by pseudoindusia; spore number 32; spores tetrahedral, tan.

##### Other specimens examined.

TAIWAN. Hsinchu County: Guanxi, Chike Mt., *P.-F. Lu 24585, 24586* (TAIF); Jianshi, *P.-F. Lu 25108* (TAIF); Pawushan, *P.-F. Lu 26666*, *26673* (TAIF); Shuitien Logging Trail, *L.-Y. Kuo 01* (TAIF). Miaoli County: Sintikusyu, komokwan, *Yaiti Simada 5175A* (HAST).

##### Distribution.

Taiwan (Fig. 4).

##### Ecology.

In shaded places, understory of evergreen broad leaf forests, below 1,000 m in elevation.

##### Etymology.

The specific epithet ‘latipinna’ refers to its wide pinnae.

##### Preliminary conservation assessment.

We investigated the distribution of *P. latipinna* Y.S.Chao & W.L.Chiou, sp. nov. in Taiwan. To date, only a few small populations are recorded. However, the available information is inadequate to support the assessment of its extinction risk. According to the IUCN (2012) criteria, the category of Data Deficient (DD) is appropriate.

**Figure 4.**
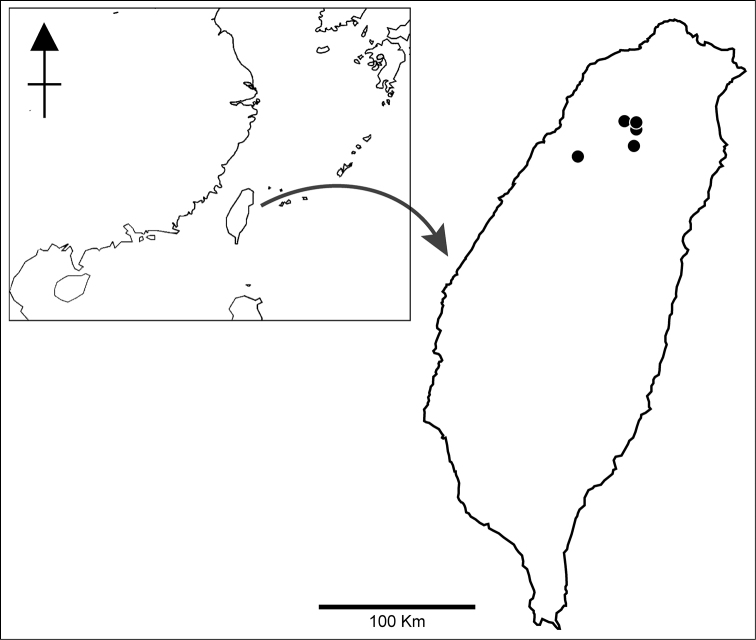
Distribution of *Pteris latipinna* Y.S.Chao & W.L.Chiou, sp. nov. (black circles) in Taiwan.

## Discussion

A new species, *P. latipinna* Y.S.Chao & W.L.Chiou, sp. nov., growing understory of forests in Taiwan was found and identified in this study. *Pteris latipinna* is the largest species among the bipinnatifid *Pteris* species with single-axis in Taiwan. There were 29 *Pteris* species recorded in the Flora of Taiwan (Shieh 1994), and several new species and new records have been recently found (Chao et al. 2013; Chao et al. 2015; Ebihara et al. 2014; Knapp 2011; Knapp and Hsu 2017). In this study, we describe one more new species, and thus in total, 36 *Pteris* species, including infraspecies, have been documented in Taiwan.

Although the ploidy of *P. latipinna* is not known, with the similar morphology and apomitic reproductive mode, it is inferred that those species possibly evolved through a complex reticulate hybridization-polyploidization speciation. Those apomicitic *Pteris* species have also been suggested with possible hybrid origins (Chao et al. 2012a; Chao et al. 2012b; Walker 1979). *Pteris latipinna* has 32 spores per sporangium, which is thought as apomictic (Chao et al. 2010; Huang et al., 2006; Nakato 1975; Walker 1979). For those species in the same clade (Clade I) of *P. latipinna*, it is reported that *P. laurisilvicola* is diploid and triploid and apomictic (Nakato 1996; Nakato and Ebihara 2016); *P. natiensis* and *P. wulaiensis* are diploid (Huang et al. 2011; Kurita 1962; Nakato and Ebihara 2016); *P. yakuinsularis* are triploid (Nakato and Ebihara 2016); *P. arisanensis* is tetrapolyploid (Tsai and Shieh 1984). Remarkably, *P. latipinna* and *P. arisanensis* have the same cpDNA characteristics although their morphologies are clearly different. They have different lamina shapes (wide ovate for *P. latipinna* vs. ovate for *P. arisanensis*) and venation (free veins in *P. latipinna* vs. costal areolae in *P. arisanensis*) (Fig. S4). Similarly, in Clade II, *P. oshimensis* does not morphologically resemble *P. fauriei* but share identical cpDNA sequences. More cpDNA and nuclear DNA markers are needed to clarify the relationships among these species in *P. fauriei* complex.

In this study, taxa in Clade I and Clade II compose *Pteris fauriei* complex because they are morphologically similar and phylogenetically close with *Pteris fauriei*. All of them are distributed in Asia, mostly in Japan and Taiwan. Interestingly, distributions of most of those species are limited: *Pteris latipinna* and *P. wulaiensis* are endemic in Taiwan; *P. boninensis*, *P. natiensis*, and *P. yakuinsularis* are endemic in Japan (Iwatsuki 1995; Shieh 1994). This pattern of distribution implies those species arose in a small area within a short time recently (Chao et al. 2014).

The traits useful for separating *P. latipinna* from the similar species are used in a key for identification of this species as shown below.

### Key for *Pteris latipinna* and related bipinnatifid *Pteris* species

**Table:** 

1	Stipes <2 mm thick	**2**
2	Pairs of lateral pinnae 4–6; basal pinnae shorter or equal to the second basal ones; pinnae narrowest at base	***P. wulaiensis***
2'	Pairs of lateral pinnae 6–11; basal pinnae longer than the second basal ones; pinnae widest at base	***P. oshimensis***
1'	Stipes 2.5–4 mm thick.	**3**
3	Laminae widely lanceolate; ratio of length to width approximately 3:2	**4**
4	Laminae bipinnatifid; the segments extending to 2/3–4/5 of the way toward the costae; venation free or with costal areolae	**5**
5	Costal areolae arched, few triangular, connective veins with free veinlets.	***P. biaurita***
5'	Costal areolae triangular or absent; if present, connected by a pair of furcated veinlets	***P. arisanensis***
4'	Laminae bipinnatisect; the segments extending almost to the costae; venation completely free, no costal areolae	**6**
6	Pinnae caudate with long tail 2–4 cm.	***P. boninensis***
6'	Pinnae acute or caudate with short tail 0.5–2 cm	**7**
7	Scales at stipe base caducous; pinnae sessile	***P. laurisilvicola***
7'	Scales at stipe base persistant; pinnae often stalked	***P. yakuinsularis***
3'	Laminae widely ovate, ratio of length to width approximately 5:4	**8**
8	Pinnae sessile except basal ones, with basal pinna-segments adnate to the rachis, pinna angle against rachis nearly 90°, incurved	**9**
9	Pinnae sometimes suddenly wider at base; segments oblong with rounded apex	***P. kawabatae***
9'	Pinnae not wider at base; segments falcate with obtuse apex	**10**
10	Pinnae nearly oblong, equally wide, 2–3 cm wide	***P. kiuschiuensis***
10'	Pinnae ovate-lanceolate to lanceolate, widest at middle, 3–6 cm wide.	**11**
11	Lateral pinnae 5–6 pairs, pinnae 3–4 cm wide, terminal pinna-segments long, >1 cm	***P. satsumana***
11'	Lateral pinnae 2–5 pairs, pinnae 3–6 cm wide, terminal pinna-segments short, <0.5 cm	***P. natiensis***
8'	Pinnae stalked to sessile, without basal pinna-segments adnate to the rachis, pinna angle against rachis 60–70°, straight	**12**
12	Basal segments of lateral pinnae triangular	***P. latipinna***
12'	Basal segments of lateral pinnae falcate	**(*P. fauriei*) 13**
13	64 spores per sporangium; laminae coriaceous	**P. fauriei var. minor**
13'	32 spores per sporangium; laminae herbaceous	**P. fauriei var. fauriei**

## Supplementary Material

XML Treatment for Pteris latipinna

## Appendix 1

**Table:** Specimen information and GenBank accession numbers.

Taxon	Specimen collection number	Collection locality	GenBank accession numbers for *rbcL*	*matK*	Herbarium for voucher specimen
*P. setulosocostulata*	*Y.-S. Chao 1146*	Taiwan	KF289634	KF289501	TAIF
*P. keysseri*	*Y.-S. Chao 1403*	Philippines	KF289640	KF289510	TAIF
*P. mucronulata*	*Y.-S. Chao 1410*	Philippines	KF289641	KF289511	TAIF
*P. pacifica*	*P.I. Forster 27643*	Australia	KF289647	KF289517	MEL
*P. kawabatae*	*Y.-S. Chao 1637*	Vietnam	KF289655	KF289525	TAIF
*P. giasii*	*C. R. Fraser-Jenkins 30176*	Bangladesh	KF289660	KF289530	TAIF
*P. kathmanduensis*	*C. R. Fraser-Jenkins FN35*	Nepal	KF289663	KF289533	TAIF
*P. otaria*	*C. R. Fraser-Jenkins FN26*	India	KF289666	KF289536	TAIF
*P. roseililacina*	*C. R. Fraser-Jenkins FN31911*	Nepal	KF289669	KF289539	TAIF
*P. biaurita*	*P.-F. Lu 17285*	Taiwan	KF289676	KF289546	TAIF
*P. argyraea*	*C. R. Fraser-Jenkins FN145*	India	KF289684	KF289554	TAIF
*P. aspericaulis*	*C. R. Fraser-Jenkins FN36*	India	KF289685	KF289555	TAIF
*P. assamica*	*C. R. Fraser-Jenkins FN5*	Nepal	KF289686	KF289556	TAIF
*P. khasiana*	*C. R. Fraser-Jenkins FN129*	India	KF289688	KF289558	TAIF
*P. praetermissa*	*C. R. Fraser-Jenkins FN64*	India	KF289692	KF289562	TAIF
*P. subindivisa*	*C. R. Fraser-Jenkins FN266*	Bhutan	KF289700	KF289570	TAIF
*P. asperula*	*Y.-C. Liu 9870*	Philippines	KF289702	KF289572	TAIF
*P. dataensis*	*Y.-C. Liu 9973*	Philippines	KF289703	KF289573	TAIF
*P. catoptera*	*G. Rouhan 301*	Madagascar	KF289714	KF289584	P
*P. humbertii*	*F. Rakotondrainibe 5965*	Madagascar	KF289718	KF289588	P
*P. confusa*	*Y.-M. Huang 20061128-A*	India	KF289726	KF289596	TAIF
*P. flava*	*M. Kurutok 23*	Sabah	KF289731	KF289601	KEP
*P. perrottei*	*C. R. Fraser-Jenkins FN215*	Nepal	KF289736	KF289606	TAIF
*P. grevilleana*	*Y.-S. Chao 770 (diploid)*	Taiwan	HM582644	KF289484	TAIF
*P. venusta*	*Y.-S. Chao 873*	Taiwan	HM582650	KF289486	TAIF
*P. longipinna*	*P.-F. Lu 11383*	Taiwan	HM582603	KF289495	TAIF
*P. laurisilvicola*	*Y.-S. Chao 1848*	Japan	KF289738	KF289608	TAIF
*P. kiuschiuensis*	*Y.-S. Chao 1852*	Japan	KF289739	KF289609	TAIF
*P. satsumana*	*Y.-S. Chao 1853*	Japan	KF289740	KF289610	TAIF
*P. oshimensis*	*Y.-S. Chao 1881*	Japan	KF289741	KF289611	TAIF
*P. yakuinsularis*	*Y.-S. Chao 1906*	Japan	KF289742	KF289612	TAIF
*P. boninensis*	*Y.-S. Chao 1941*	Japan	KF289743	KF289613	TAIF
*P. natiensis*	*Y.-S. Chao 1835*	Japan	KF289744	KF289614	TAIF
*P. arisanensis*	*Y.-S. Chao 1621*	Vietnam	KF289677	KF289547	TAIF
*P. latipinna*	*P.-F. Lu 24585*	Taiwan	MF416317	MF416323	TAIF
*P. latipinna*	*P.-F. Lu 25108A*	Taiwan	MF416318	MF416324	TAIF
*P. latipinna*	*P.-F. Lu 25108B*	Taiwan	MF416319	MF416325	TAIF
*P. wulaiensis*	*P.-F. Lu 26667-1*	Taiwan	MF537503	MF537504	TAIF
P. fauriei var. minor	*Y.-S. Chao 2078*	Taiwan	MF416320	MF416327	TAIF
P. fauriei var. fauriei	*Y.-S. Chao 2083*	Taiwan	MF416321	MF416328	TAIF
*P. latipinna*	*Y.-S. Chao 2092*	Taiwan	MF416322	MF416326	TAIF

## Supplementary materials

Supplementary material 1 Figure S1. Data type: JPEG image file Explanation note: Type material of Pteris fauriei Hieron. var. fauriei in B (B20012819). This dataset is made available under the Open Database License (http://opendatacommons.org/licenses/odbl/1.0/). The Open Database License (ODbL) is a license agreement intended to allow users to freely share, modify, and use this Dataset while maintaining this same freedom for others, provided that the original source and author(s) are credited.Yi-Shan Chao, Atsushi Ebihara, Wen-Liang Chiou, Yao-Moan Huang

Supplementary material 2 Figure S2. Data type: JPEG image file Explanation note: Type material of Pteris fauriei var. minor Hieron. in B (*U. Fauriei 685*, B200128109). This dataset is made available under the Open Database License (http://opendatacommons.org/licenses/odbl/1.0/). The Open Database License (ODbL) is a license agreement intended to allow users to freely share, modify, and use this Dataset while maintaining this same freedom for others, provided that the original source and author(s) are credited.Yi-Shan Chao, Atsushi Ebihara, Wen-Liang Chiou, Yao-Moan Huang

Supplementary material 3 Figure S3. Data type: JPEG image file Explanation note: Holotype of *Pteris natiensis* Tagawa in KYO (*G. Koidzumi s.n.* Aug. 3, 1922). This dataset is made available under the Open Database License (http://opendatacommons.org/licenses/odbl/1.0/). The Open Database License (ODbL) is a license agreement intended to allow users to freely share, modify, and use this Dataset while maintaining this same freedom for others, provided that the original source and author(s) are credited.Yi-Shan Chao, Atsushi Ebihara, Wen-Liang Chiou, Yao-Moan Huang

Supplementary material 4 Figure S4. Data type: JPEG image file Explanation note: Holotype of *Pteris arisanensis* Tagawa in KYO (*U. Fauriei 603*). This dataset is made available under the Open Database License (http://opendatacommons.org/licenses/odbl/1.0/). The Open Database License (ODbL) is a license agreement intended to allow users to freely share, modify, and use this Dataset while maintaining this same freedom for others, provided that the original source and author(s) are credited.Yi-Shan Chao, Atsushi Ebihara, Wen-Liang Chiou, Yao-Moan Huang

Supplementary material 5 Figure S5. Data type: JPEG image file Explanation note: Holotype of *Pteris wulaiensis* C.M. Kuo in TAI (*S.-J. Moore4383*, TAI283138). This dataset is made available under the Open Database License (http://opendatacommons.org/licenses/odbl/1.0/). The Open Database License (ODbL) is a license agreement intended to allow users to freely share, modify, and use this Dataset while maintaining this same freedom for others, provided that the original source and author(s) are credited.Yi-Shan Chao, Atsushi Ebihara, Wen-Liang Chiou, Yao-Moan Huang
