# Supplementary figures and images for: Pteris latipinna sp. nov. (Pteridaceae), a new species segregated from Pteris fauriei
Source: PhytoKeys. 2017 Aug 31;(85):95–108. doi: 10.3897/phytokeys.85.14884 (PMC5624215; doi:10.3897/phytokeys.85.14884)

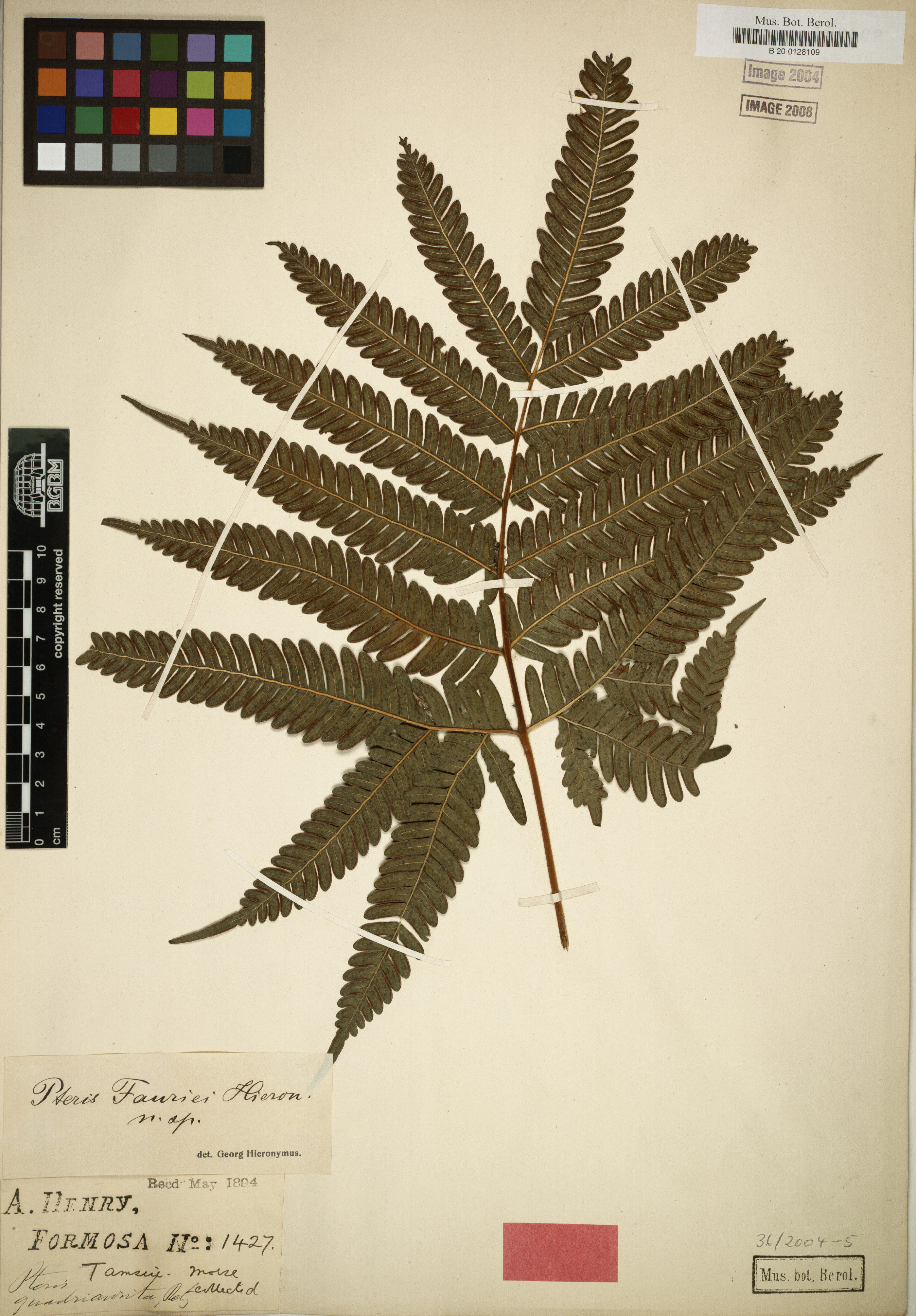

Supplement: Supplementary material 1 — Figure S1. [file phytokeys-85-095-s001.jpg]

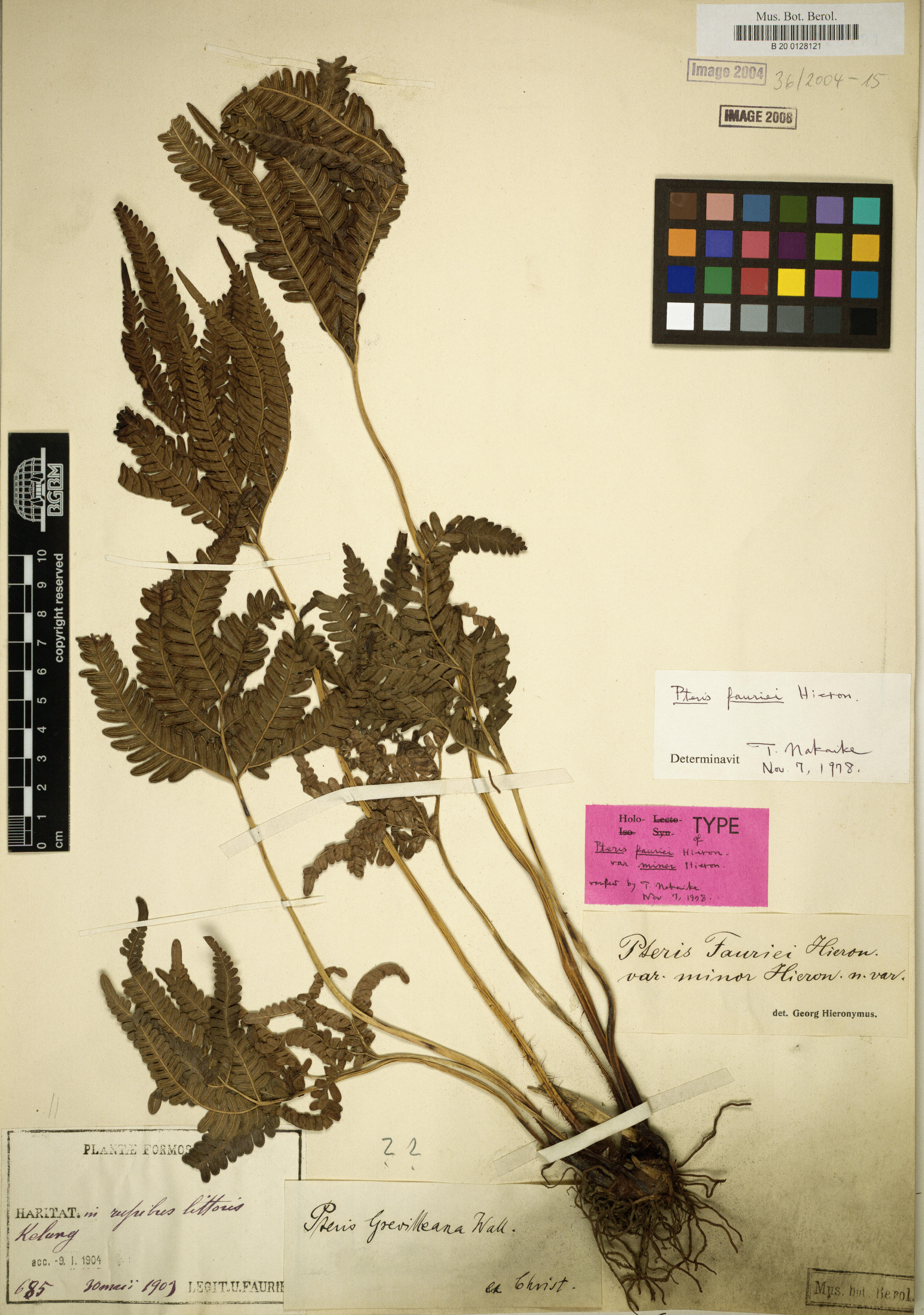

Supplement: Supplementary material 2 — Figure S2. [file phytokeys-85-095-s002.jpg]

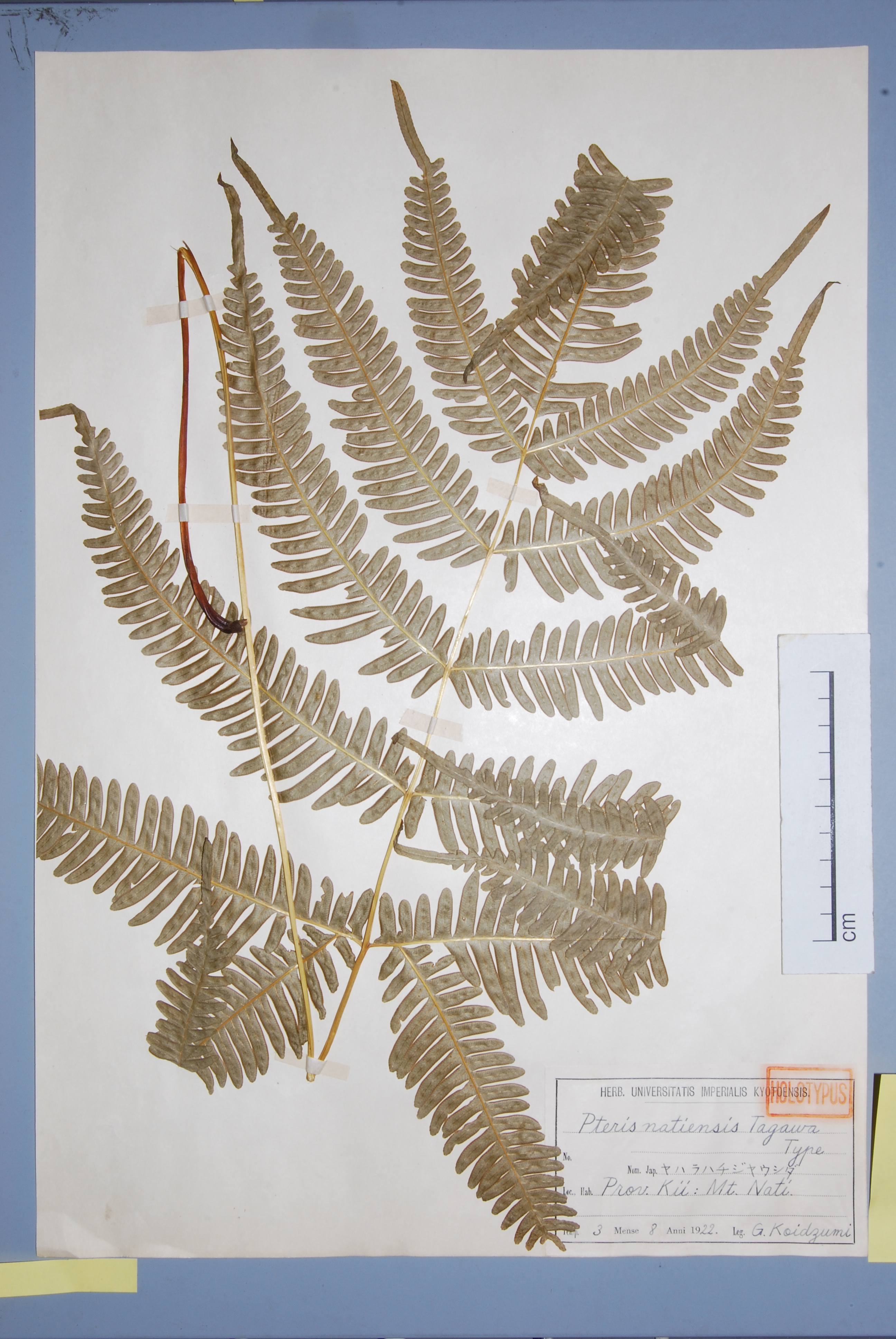

Supplement: Supplementary material 3 — Figure S3. [file phytokeys-85-095-s003.jpg]

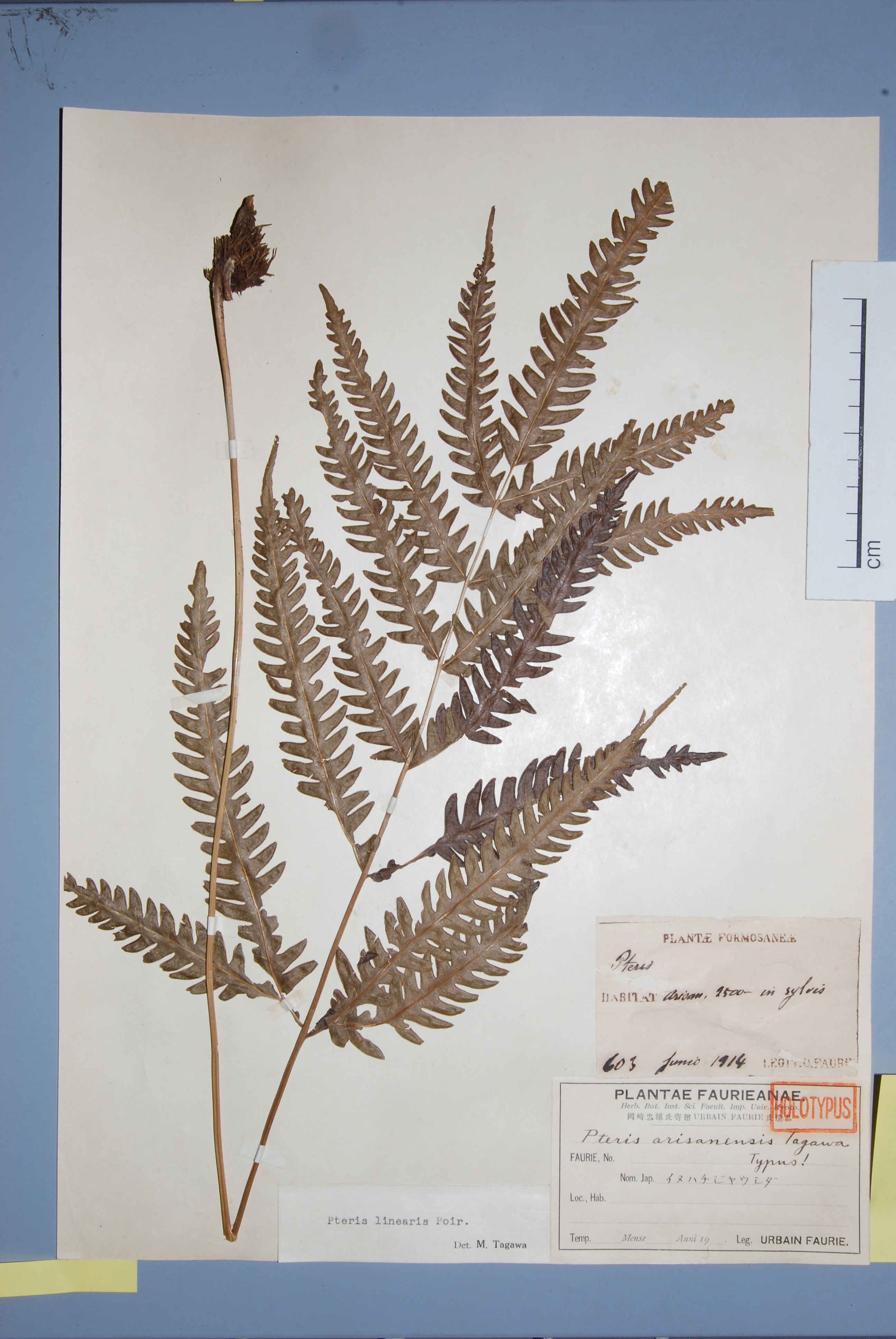

Supplement: Supplementary material 4 — Figure S4. [file phytokeys-85-095-s004.jpg]

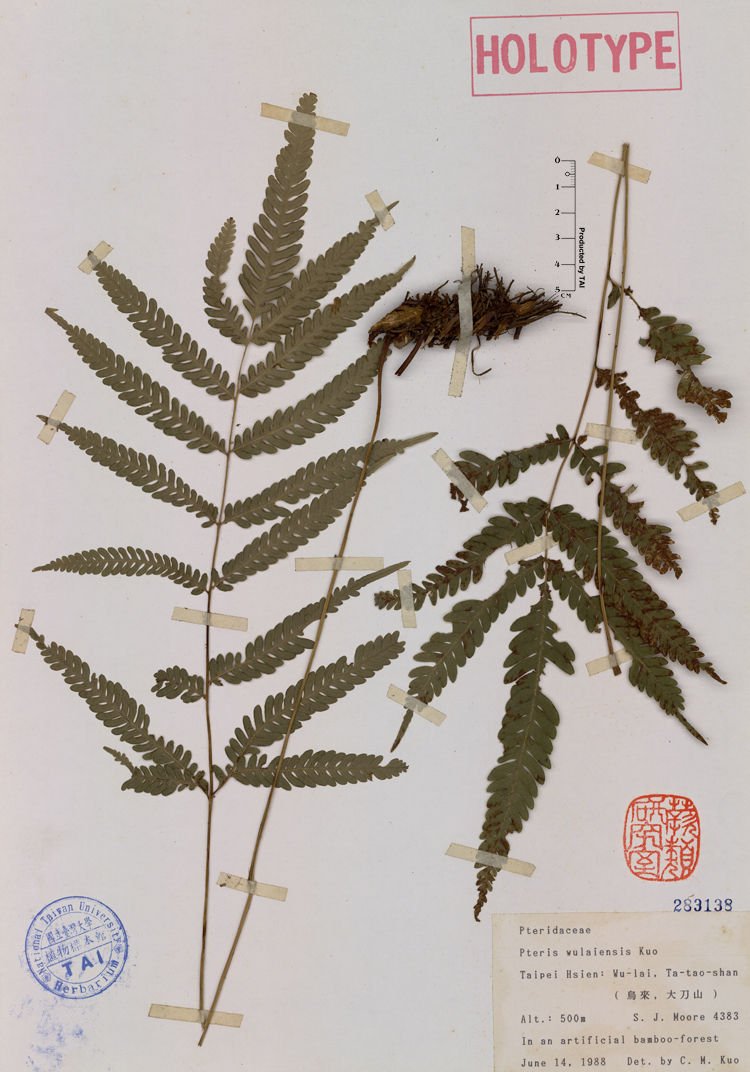

Supplement: Supplementary material 5 — Figure S5. [file phytokeys-85-095-s005.jpg]
